# A Multimedia Child Developmental Screening Checklist: Design and Validation

**DOI:** 10.2196/jmir.6249

**Published:** 2016-10-24

**Authors:** Hsin-Yi Kathy Cheng, Li-Ying Chen, Chih-Hsiu Cheng, Yan-Ying Ju, Chia-Ling Chen, Kevin C Tseng

**Affiliations:** ^1^Graduate Institute of Early InterventionCollege of MedicineChang Gung UniversityTaoYuanTaiwan; ^2^Department of Physical Medicine and RehabilitationChang Gung Memorial HospitalTaoYuanTaiwan; ^3^Department of Physical Therapy and Graduate Institute of Rehabilitation ScienceCollege of MedicineChang Gung UniversityTaoYuanTaiwan; ^4^Department of Adapted Physical EducationCollege of Physical EducationNational Taiwan Sport UniversityTaoYuanTaiwan; ^5^Department of Industrial DesignCollege of ManagementChang Gung UniversityTaoYuanTaiwan

**Keywords:** child development, multimedia, screening, usability, Web-based, disability

## Abstract

**Background:**

Identifying disability early in life confers long-term benefits for children. The Taipei City Child Development Screening tool, second version (Taipei II) provides checklists for 13 child age groups from 4 months to 6 years. However, the usability of a text-based screening tool largely depends on the literacy level and logical reasoning ability of the caregivers, as well as language barriers caused by increasing numbers of immigrants.

**Objective:**

The objectives of this study were to (1) design and develop a Web-based multimedia version of the current Taipei II developmental screening tool, and (2) investigate the measurement equivalence of this multimedia version to the original paper-based version.

**Methods:**

To develop the multimedia version of Taipei II, a team of experts created illustrations, translations, and dubbing of the original checklists. The developmental screening test was administered to a total of 390 primary caregivers of children aged between 4 months and 6 years.

**Results:**

Psychometric testing revealed excellent agreement between the paper and multimedia versions of Taipei II. Good to excellent reliabilities were demonstrated for all age groups for both the cross-mode similarity (mode intraclass correlation range 0.85-0.96) and the test-retest reliability (*r*=.93). Regarding the usability, the mean score was 4.80 (SD 0.03), indicating that users were satisfied with their multimedia website experience.

**Conclusions:**

The multimedia tool produced essentially equivalent results to the paper-based tool. In addition, it had numerous advantages, such as it can facilitate active participation and promote early screening of target populations.

**ClinicalTrial:**

Clinicaltrials.gov NCT02359591; https://clinicaltrials.gov/ct2/show/NCT02359591 (Archived by WebCite at http://www.webcitation.org/6l21mmdNn)

## Introduction

Identifying disability early in life confers long-term benefits for children, particularly those with special needs. To detect those who need help early, judicious use of practical and reliable standardized screening tools is of great importance. Most children who are diagnosed with disabilities are not identified before entering school [1,2]. Their developmental problems are often associated with poor health, low school performance, high in-grade retention, and special education placement [3]. Early intervention for children not only enhances child developmental outcomes, but also improves parents’ ability to care for their children and increase family quality of life [4].

Clinically, disorders such as cerebral palsy and profound intellectual disability are clearly recognizable. However, subtle disabilities, such as mild intellectual disability and learning disabilities, can often escape detection in the early years of life despite frequent well-child visits [5,6]. Physicians generally acknowledge the importance of screening for developmental disabilities, but most of them rely on clinical judgment and milestones instead of standardized screening instruments [3]. A survey study conducted in 2011 reported that among 1821 pediatricians, less than half screened patients younger than 36 months with formal screening tools [7]. The main barriers cited in preventing the use of such tools included time limitations, lack of staff to perform screening, and inadequate reimbursement. Conducting formal neurodevelopmental assessments by using standardized tests presents numerous operational difficulties.

Therefore, researchers have searched for alternatives, such as using parent-reported data [8]. Primary caregivers typically know their children better than their physicians and are able to identify most developmental problems. The Taipei City Child Development Screening tool, second version (Taipei II) is a valid screening tool that was developed and funded by the Taiwan health authority. It provides checklists for 13 child age groups from 4 months to 6 years (4, 6, 9, 12, 15, 18, 24, 30, 36, 42, 48, 60, and 72 months), with 11 to 13 behavior/skill items related to gross/fine motor, cognition, language/communication, and emotion/social areas easily observed or elicited by the child’s caregiver. Methodology research testing the reliability and validity of the Taipei II, using a sample of 506 children aged 5.5 to 35.5 months, was performed. To simulate the clinical situation for validity analysis, the Taipei II checklist was filled out by one parent or a main caregiver at clinics after explaining the purpose of this study and the rating principles. The results revealed that the sensitivity ranged from 0.85 to 1.00 and the specificity ranged from 0.82 to 1.00 if the cutoff was set at “failure to pass more than one item.” For test-retest reliability, the data of the Taipei II were collected twice within a time interval of 1 week. Significant reliability coefficient of the total score was reported. In addition, the checklist also demonstrated a significant and moderate-to-high screening accuracy (*P*<.05) for each age-appropriate checklist via the receiver operating characteristics curve [9]. Taipei II is typically delivered to caregivers during well-child visits, which is passive and often fails to deliver if the caregivers miss the visit. In addition, Taipei II is text-based, and the usability of a text-based screening tool largely depends on the literacy level and logical reasoning ability of the caregivers, as well as language barriers because of increasing numbers of immigrants [10].

To help caregivers comprehend textual information, illustrated medical instructions and education tools have been increasingly used in recent years [11,12]. Illustrations have various functions: they support comprehension of textual content, provide a clear structural framework, help to clarify difficult passages, direct users’ attention to the material, and enhance enjoyment [13]. Recently, multimedia has been considered to more effectively exert vision-mediated effects; hence, it has also become a trend in promoting learning and comprehension [14,15], including a mixture of static or dynamic illustrations and sound effects, particularly with computers. Use of computers and mobile phones is now widespread and users can access information actively and easily by using these devices. Therefore, a Web-based multimedia system would facilitate active participation and assist in comprehension of the target contents. The purposes of this study were to (1) design and develop a Web-based multimedia version of the Taipei II developmental screening tool and (2) investigate the psychometric properties of this multimedia mode compared to the original text version.

## Methods

**Figure 1:**
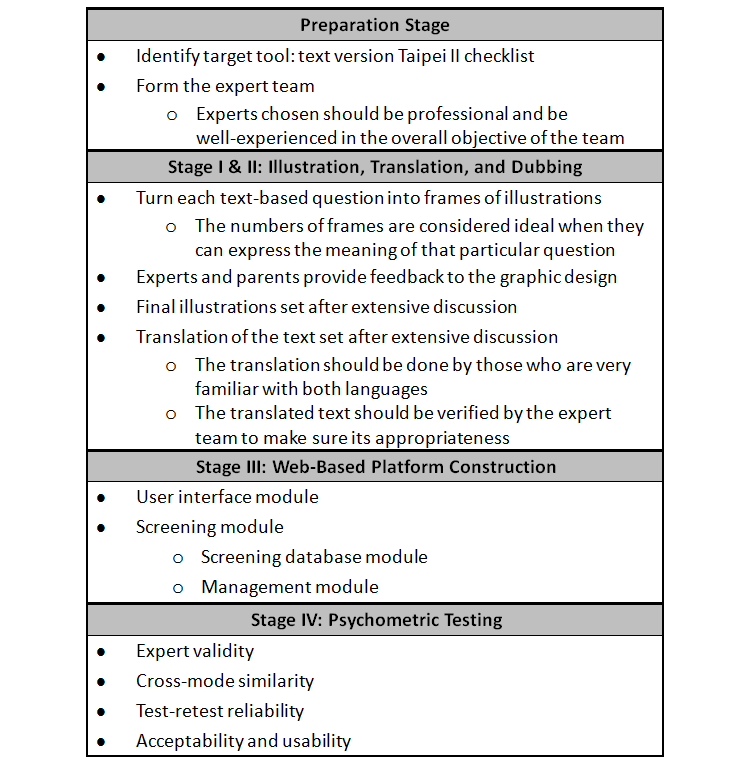
Flowchart depicting the design stages of the multimedia system.

This study was conducted in four stages: the first stage involved illustrating each text-based question, the second stage was the translation and dubbing, the third stage was the Web-based system construction, and the fourth stage was testing the psychometric properties of the final multimedia system and comparing them with the original paper version (Figure 1). These four stages also represented the key processes when turning a paper-and-pencil checklist to a multimedia Web-based format. Other detailed considerations, such as the ideal amount of illustrations and the particular backgrounds of the experts, were case-specific.

## Participants

This study involved clinics and communities located in different areas of Northern Taiwan, representing the social and cultural contexts in the region. Three pediatric and family physician clinics participated. Participants recruited through communities were referred from six local public health centers. Primary caregivers of children aged between 4 months and 6 years were included to perform the developmental screening test. Considering that one of the primary goals of this study was the validation of a multimedia version of Taipei II, participants were excluded if they had any visual, auditory, or other deficits that would hinder them in operating the Web-based computer interface. No other exclusion criteria were applied. A total of 390 participants (104 men, 286 women) with a mean age of 33.35 (SD 6.71, range 23-70) years joined this study. Each age group consisted of 30 participants. The mean ages of their children were 29.8 (SD 20.6, range 4-84) months.

### Design Stages

#### Preparation Stage

Taipei II was identified as the target tool to transform. A task force of experts was responsible for the developmental process. The task force primarily consisted of child development, rehabilitation, and graphic/Web design professionals, reflecting a broad array of backgrounds, perspectives, and expertise that enriched the study (Table 1). The developmental process of the multimedia version of Taipei II lasted approximately 10 months.

**Table 1:** Details of the expert team.

Member ID	Profession	Years of experience	Role on the team
1	Child development; rehabilitation; evidence-based research	22	Multimedia system design and validation; website conceptualization and design; coordinated and supervised study progress; supervised data collection at clinical sites; data analysis
2	Child development; nursing	5	Multimedia system design; website conceptualization and design; coordinated and supervised study progress; data collection and analysis
3	Child development; rehabilitation	21	Website conceptualization and design; system validation
4	Child development; physical therapy	16	System validation
5	Child development; physical therapy	12	System validation
6	Pediatrics; rehabilitation	20	System validation
7	Graphic design; Web design	20	Multimedia system design; website conceptualization and design; coordinated and supervised study progress
8	Graphic design; Web design	6	Multimedia system design; website conceptualization and design; character design and drawing
9	Graphic design	5	Character design and drawing
10	Graphic design	19	Character design and drawing

#### Stages I and II: Illustration, Translation, and Dubbing

The goal of this stage was to turn each text-based question into at least four illustrations to be displayed in sequence on the screen of a mobile device or computer. A subset of the task force members formed a discussion group that prepared subsequent iterations of the design, including illustrations and translations, and distributed them to the full task force for review and feedback. Because this screening tool is applied to children with a wide age range, and children of different ages differ in appearance, the graphic designer was first provided with pictures of children for each age group. Thus, the designer knew what the characters should look like at different developmental stages. Efforts were then made to ensure that the graphic designer understood the meanings of the questions to assist in composing the illustrations. Two experts were in charge of this process. The translation and voice recordings for dubbing were also checked extensively.

During this period, drafts of each illustrated checklist were distributed to volunteer parent groups. These parents provided feedback regarding the illustrations and indicated whether they were clear and attractive. The illustrations were then modified accordingly. This version was reevaluated by the task force. The translations were also checked by the task force through extensive discussion before dubbing. The final illustrations were dubbed in both English and Chinese.

#### Stage III: Web-Based Platform Construction

**Figure 2:**
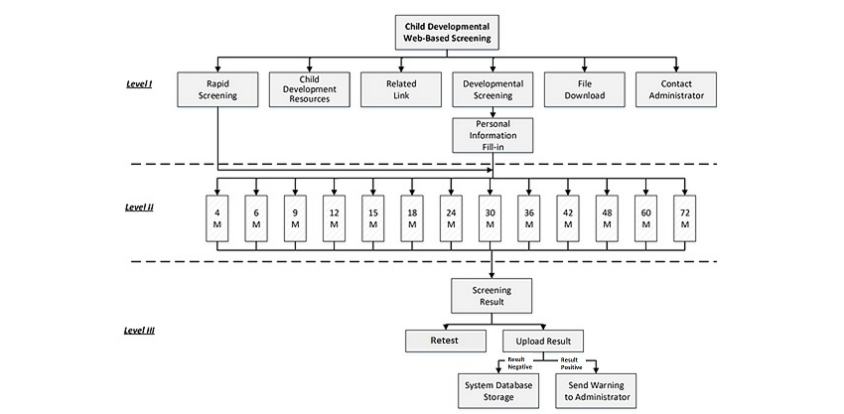
The website interface of the child developmental screening system.

The proposed system is a Web-based system that fulfills the needs of early childhood developmental screening by involving caregivers, clinical professionals, and government officials over the Internet. It is a professional child screening system presented in a playful style. In total, 144 checklists were developed for 13 different age groups (4, 6, 9, 12, 15, 18, 24, 30, 36, 42, 48, 60, and 72 months). The overall flow of this multimedia system is shown in Figure 2. It represents the interface of the website. Level I consists of information regarding child development and related resources. If the “rapid screening” or “developmental screening” icon is clicked or touched, the user enters Level II, where the screening takes place. Once done with the screening process, the user enters Level III for screening results. The user can also choose whether to retake the test or upload the result to the cloud for data storage and/or warning the administrator.

**Figure 3:**
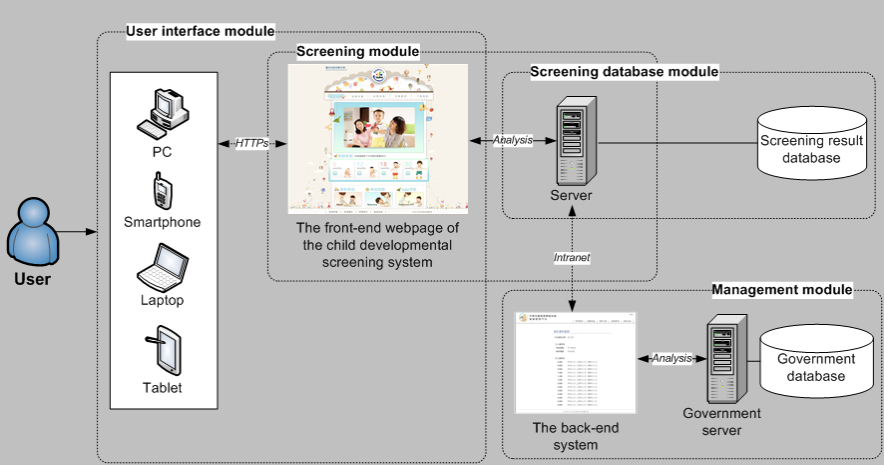
System framework.

The child developmental screening system framework is depicted in Figure 3. The Web-based system can be used with Hypertext Transfer Protocol online encryption to enhance information safety. In addition, the back-end system enables government officials to monitor data such as users’ health-related information and their screening results.

The system contains four major modules:

1. User interface module: this module provides a Web-based interface for users to use the child screening service on different devices, such as mobile phones or PCs, provided that Internet access is available.

2. Screening module: this module contains the front-end webpage of the child developmental screening system. A server that included information such as Web interface data, user interaction data, and health information browsing history was linked to this screening module. It also analyzes the screening results collected from the users. When users take the screening test on the system, it automatically processes the input data and determines whether the child passes the test for his or her developmental age. The analyzed data are then saved in the screening database for future search and use.

3. Screening database module: this module preserves data collected from users’ screening tests. The screening results can also be transmitted to the Department of Health of the Taipei City Government or exported in .csv format for future use.

4. Management module: the government database manager receives users’ screening results from the back-end system management platform. Experts can interpret the screening results and provide relevant suggestions or assistance.

The service engine of the system platform can be widely applied to all organizations and users. In addition, the complete multimedia checklists were provided on the Internet and can be used in various browsers. The front-end webpage includes not only the developmental checklists, but also reference sources and information regarding healthy child development, general health information for children, and early intervention and education resources. The back-end control panel can be accessed by registered health care consultants. When a positive screening result raises an alarm, attention can be focused on the child and the consultants can make further contact with the caregivers of that child by phone or mail. Follow-up, such as at-home interviews or physician visits, should be provided on the basis of the initial screening results in addition to the phone conservation, if possible.

#### Stage IV: Psychometric Testing

##### Expert Validity

The complete multimedia version of Taipei II was checked for measurement equivalence to the text-based paper-and-pencil version by calculating expert validity. Task force agreement was measured using a four-point Likert-type rating scale [16] (1=not relevant, 2=somewhat relevant, 3=quite relevant, and 4=very relevant). This proportional agreement procedure, calculated using a content validity index (CVI), allows two or more raters to independently review and evaluate the relevance of the illustrations and original text. Multiple iterations of illustration, translation, and dubbing were obtained. The stability of their agreements was determined on the basis of these results.

The final multimedia version was used for cross-mode similarity, test-retest reliability, acceptability, and usability tests. The study protocol was explained to the participants and they were asked to provide informed consent. They were also informed that the data might be made available to government officials. Baseline demographics of the participants and their children were collected to ensure their eligibility. The participants were asked to complete the developmental checklists that matched the age of their children. This study protocol was approved by the Institutional Review Board of Chang Gung Medical Hospital, TaoYuan, Taiwan.

##### Cross-Mode Similarity

To determine the measurement equivalence between the two versions, a crossover design was implemented. Participants were randomly assigned to complete either the paper version or the multimedia version of Taipei II for the first administration, and the other version for the second administration. A 2-week washout period was enforced between administrations to minimize the carryover effects from the first administration. The cross-mode similarities between checklists for the different age groups were calculated as: ([test number]–[the number of differences])×100%.

##### Test-Retest Reliability

The test-retest reproducibility was determined for both the paper and multimedia versions. Two weeks after the end of the previously mentioned crossover study, the participants again completed the same version of the developmental checklist they completed in the second administration during the crossover period. The authors tallied the participants’ responses and calculated the similarity for each age group.

##### Usability and Acceptability of the Web-Based Platform

Combined quantitative and qualitative methods were used to evaluate the usability and acceptability of the Web-based multimedia platform. A five-point Likert scale was used to measure the usability of this multimedia system. The testing items included the screen layout, information displayed on the screen, arrangement of information on the screen, clarity of the expression, ease of navigation, and overall experience with the website. A score of 5 meant most comfortable or extremely satisfied for that particular item. The mean score for the usability items was calculated. Acceptability was assessed using quantitative data regarding whether the participants preferred the Web-based multimedia checklists or the paper-and-pencil checklists or had no specific preference based on its usefulness. The Web-based method was considered acceptable if more than 50% of the parents preferred it to the paper instrument or had no preference [17]. Qualitatively, a semistructured interview was administered. The interviewer asked the parents’ opinions regarding the operation of the website, the quality and layout of the pictorial designs, any improvement needed, or suggestions they had for the multimedia version. The postsurvey interview took no more than 5 minutes.

## Results

Throughout the developmental process, there were multiple iterations of illustrations, translation, and dubbing, and the expert validities were obtained twice: the first and the last iterations. The calculated content validities for the two major revisions for each age group are listed in Table 2 for both illustrations and translation/dubbing. The experts achieved a consensus for the final revision. For the overall cross-mode similarity, the mean score for the paper and the multimedia version was 0.94 (Table 2). For test-retest reliability, the mean score was high (*r*=.93) (Table 2).

**Table 2:** Expert validity for the first and final iterations, cross-mode similarity for the paper and the multimedia version, and test-retest reliability for the multimedia version.

Psychometrics		Overall	Child age (months)												
			4	6	9	12	15	18	24	30	36	42	48	60	72
**Expert validity, CVI**															
	First iteration		0.97	0.95	0.99	0.69	0.95	0.85	0.96	0.85	0.90	0.81	0.90	0.86	0.86
	Final iteration		1.00	1.00	1.00	0.95	1.00	0.91	1.00	0.92	1.00	1.00	1.00	1.00	1.00
Cross-mode similarity, mode intraclass correlation		0.94	0.94	0.88	0.89	0.85	0.89	0.95	0.89	0.96	0.95	0.96	0.92	0.94	0.96
Test-retest reliability, *r*		.93	.95	.89	.90	.87	.91	.95	.91	.96	.95	.96	.92	.95	.97

**Table 3:** Usability test for the multimedia website (N=390).

Usability items	Score, n					Usability score, mean (SD)
	1	2	3	4	5	
Screen layout	0	0	1	58	331	4.85 (0.37)
Information displayed on the screen	0	0	2	88	300	4.76 (0.44)
Arrangement of information on the screen	0	0	2	87	301	4.77 (0.44)
Ease of navigation	0	0	0	75	315	4.81 (0.39)
Overall experience with the website	0	0	0	77	313	4.80 (0.40)
Mean usability score for all items						4.80 (0.03)

### Usability and Acceptability of the Web-Based Multimedia Platform

Quantitatively, the mean usability score for all the test items was 4.80 (SD 0.03), indicating that users were satisfied with their multimedia website experience. The individual scores for testing items are depicted in Table 3. In addition, 97.9% (382/390) of participants preferred the Web-based multimedia version to the paper version, less than 0.8% (3/390) preferred the paper version, and 1.3% (5/390) expressed no preference. In total, 99.2% (387/390) preferred the Web-based multimedia version or had no preference, strongly supporting its acceptability. Qualitatively, participants suggested adding more illustrations or even animation to certain questions to make the checklist clearer and more attractive.

## Discussion

Early identification of developmental delays is essential for optimal early intervention. Children with subtle developmental problems often remain unidentified as such; therefore, regular screening is of great importance. Typical text-based screening can overlook respondents with low literacy and those whose first language differs from the text. In addition, text-based screening tools lose users’ attention easily. This study successfully transformed the text-based Taipei II into a multimedia version, and the two modes of administration produced essentially equivalent results. Based on feedback from the participants, the Web-based multimedia mode demonstrated higher acceptability and accessibility than the original version.

Compared with text-based instructions, illustrations and spoken information promote clearer understanding, particularly among people with limited literacy skills or cognitive impairment [11,12]. They promote text comprehension through two effects: increasing motivation and deepening elaboration [11]. Research on learning with text and pictures has yielded numerous recommendations on how to design effective multimedia instruction [14]. It has been proposed that adding visualizations to text (ie, the multimedia principle), using spoken rather than written text to accompany visualizations (ie, the modality principle), and using spoken rather than written and spoken text (ie, the redundancy principle) aid learning. These principles also reflected the needs of our parent groups. Parents’ concerns and suggestions included the attractiveness of the designed characters, the clearness of the dubbing contents, and whether the contents were easy to comprehend. Our multimedia developmental screening system consisted of illustrations and dubbings that were evaluated by both the expert teams and the intended users, thereby facilitating clearer understanding for the caregivers.

**Figure 4:**
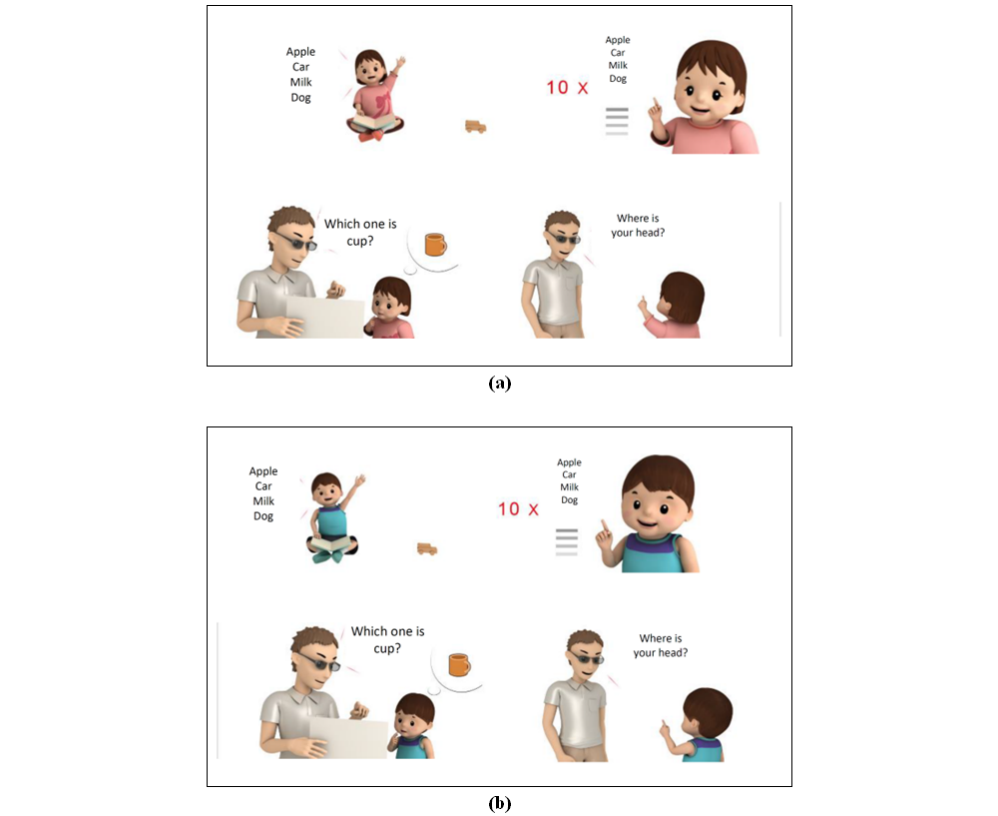
Screening checklists for age 24 months (questions 3-5): (a) girl’s version, (b) boy’s version.

Our results revealed a satisfactory overall similarity of 0.94 between the two versions. This result was achieved through close cooperation among the experts. During the developmental process, the expert team strived to make the presentation of the multimedia system vivid and lively. Research has indicated that positive emotional feelings play a critical role in multimedia learning and should be considered when designing multimedia materials [18]. Therefore, the graphic designers used warm colors and smooth shapes for the child characters (eg, a pink dress for girls and a blue shirt for boys) and their surrounding environment (Figure 4). Age-appropriate appearance (eg, younger children with proportionally larger heads) was also considered during the illustration process. In addition, sound has also been demonstrated to affect comprehension [19]. The team chose a calm female voice to dub each item in the checklists. All these efforts were intended to accurately convey the meaning of the original text-based version and to maintain caregivers’ attention.

Physicians generally acknowledge that screening for developmental disabilities is crucial; however, because they are often overwhelmed with patients and constrained for time, few use standardized screening instruments [5,6]. With the current Web-based multimedia screening tool, children can be evaluated by their caregivers. Coupled with the clinical judgment of physicians, this screening tool can considerably improve the rates of appropriate screening and time to diagnosis of children with developmental delay. Hence, such children can be directed to appropriate early intervention services in a timely manner. Still, necessary cautions should be made when using this promising multimedia screening tool. Future studies can focus on other types of reliability tests, such as the stability (ie, the performance agreement over months or years) and other types of validity tests, such as the accuracy of this multimedia system. Alternative screening with proven accuracy, such as Bayley Scales or Vineland Adaptive Behavior Scales, can also be used for developmental screening and their multimedia version can be built in reference to the current process.

The complete developmental process of this multimedia system can be modeled when one is intended to design an effective multimedia tool. The methods and considerations within the four stages in this study (ie, the design of illustration, the translation and dubbing, the Web-based system construction, and the psychometric properties evaluation) can serve as guidelines during the construction of many multimedia system platforms.

In summary, the results indicate that the Web-based multimedia checklists successfully retain the psychometric properties of the original paper-based tool. These findings also support the usefulness of Web-based multimedia checklists as an appropriate development screening tool for children aged between 4 months and 6 years. With the added illustrations and dubbing, the checklists became clearer and more attractive. Moreover, the Web-based tool is easily accessed, facilitating active participation. The team’s next step is promoting this multimedia checklist through the broadcasting media or advertisements in health care agencies, pediatric clinics, public areas, and other related organizations.

## Multimedia Appendix 1

Developmental e-screening leaflet.

## Abbreviations

CVI: content validity index
Taipei II: Taipei City Child Development Screening tool, second version
