# Supplementary material for: A Multimedia Child Developmental Screening Checklist: Design and Validation
Source: J Med Internet Res. 2016 Oct 24;18(10):e277. doi: 10.2196/jmir.6249 (PMC5099499; doi:10.2196/jmir.6249)
Supplement: Supplementary file 1 [file jmir_v18i10e277_app1.pdf]

Convenient Developmental E-Screening Saves Your Time and Efforts
Early Screening: The Golden Period of Child Development
http://e-screening.health.gov.tw

Taipei City Child Developmental E-Screening Interactive Website

About the Website

The site is to screen children of 6 years old and under. Visit it via a computer or smartphone at http://e-screening.health.gov.tw (Taipei City Child Developmental E-Screening Interactive Website)→ start screening→ type in age (13 age groups in total, ex. 6 months, 12 months, 3 years or 6 years old)→ respond to the questions→ check results→ screening completed, fast and easy. Upload the results and if there is anything untypical, professional and caring support will be provided.

Features

(1) Google "[illegible]" (Taipei City Child Developmental E-Screening Interactive Website)

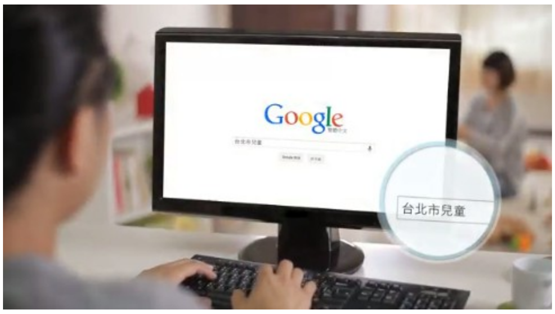

(2) A introductory video clip helps you learn quickly how to use the site

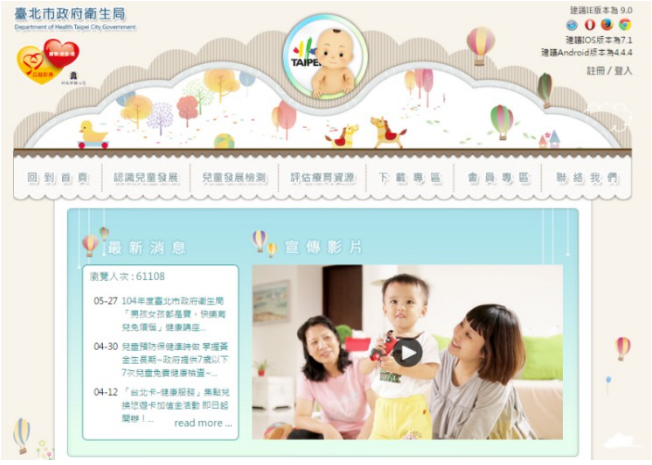

Sections: Homepage, What is Child development, Child Developmental Screening, Sources for Early Intervention, File Downloads, Member Account, Contact Us

(3) Screening questions with colorful images targeting 13 age groups

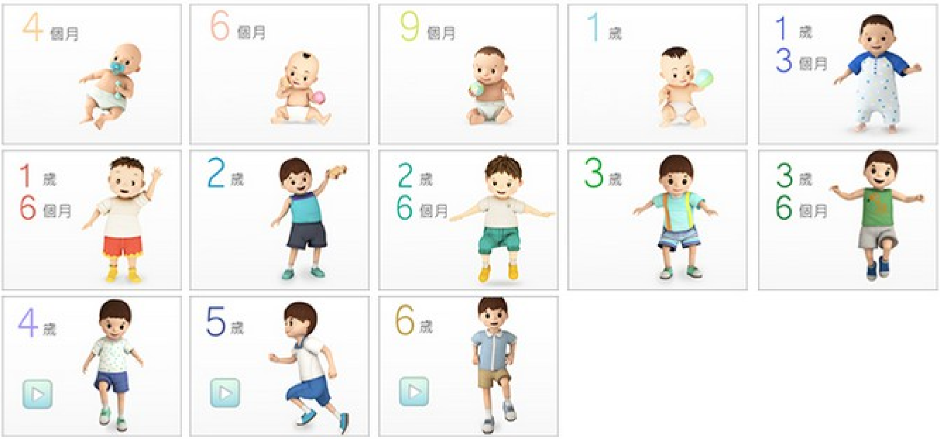

Ages include:

4 months, 6 months, 9 months, 12 months, 15 months, 1.5 years, 2 years, 2.5 years, 3 years, 3.5 years, 4 years, 5 years, 6 years

Choose or enter the date of birth and the gender of your child. For example, the first question for a 2-year-old: Can your baby kneel down or bent down to pick things up from the floor and stand back up with things still in hand?

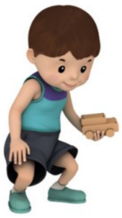

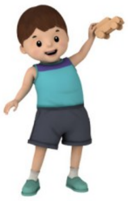

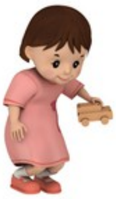

Girl’s version

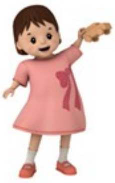

Boy’s version

Choose your preferred language

(5) Active Follow-Up: In case of possible developmental delay, professional support will be provided.

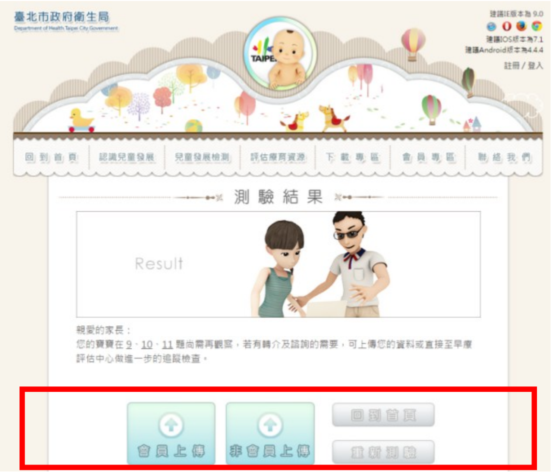

Member uploads, Non-member uploads, Back to Home, Test again

(6) Your Account/ Customize Services: members will be able to receive the latest news and follow the growth and development of their child through account history anytime.

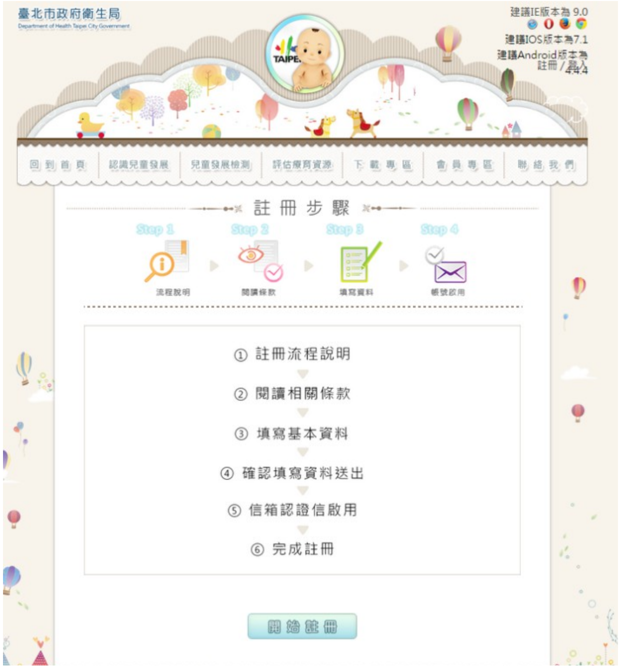

How to Create an Account, Membership Policy, Personal Information, Check and Submit, Confirm Your Email Address, Registration Successful, Create an Account

HOW TO USE THE WEBSITE

STEP1 Visit the site at: http://e-screening.health.gov.tw/

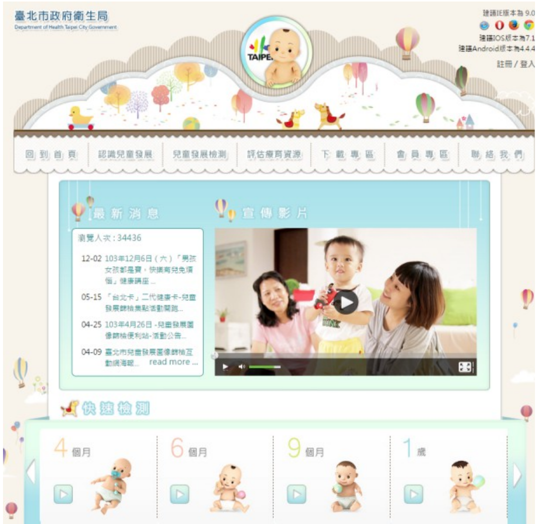

STEP2 Start screening

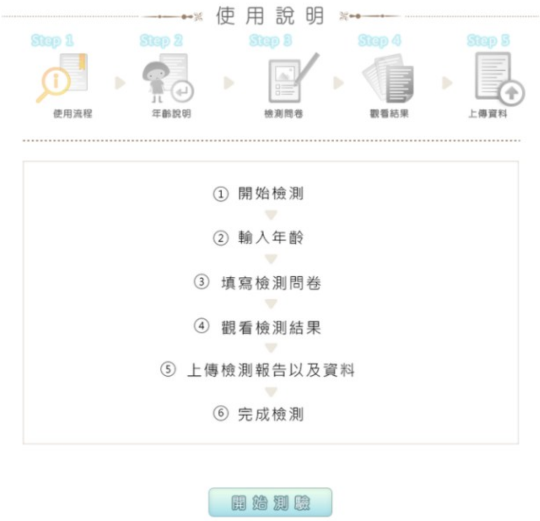

Start Screening, Enter Age, Answer the Questions, Check the Results, Upload Results and Data, Screening Completed

STEP3 Enter background information of your child (gender, date of birth, height and weight)

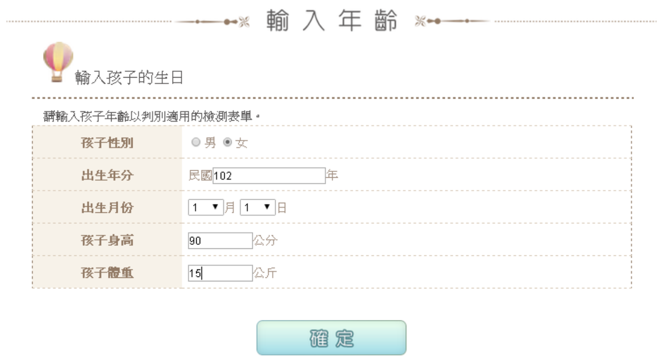

Gender, Year of Birth, Month of Birth, Height, Weight, Submit

STEP4 Turn on Your Speaker, Choose a Preferred Language

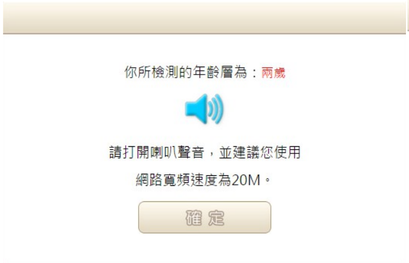

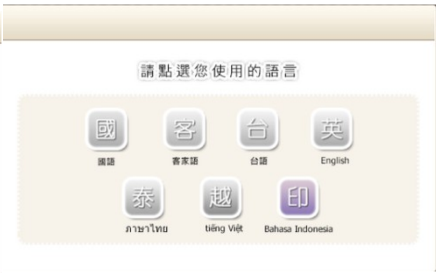

20M broadband internet connection is recommended.

STEP5 Start Screening

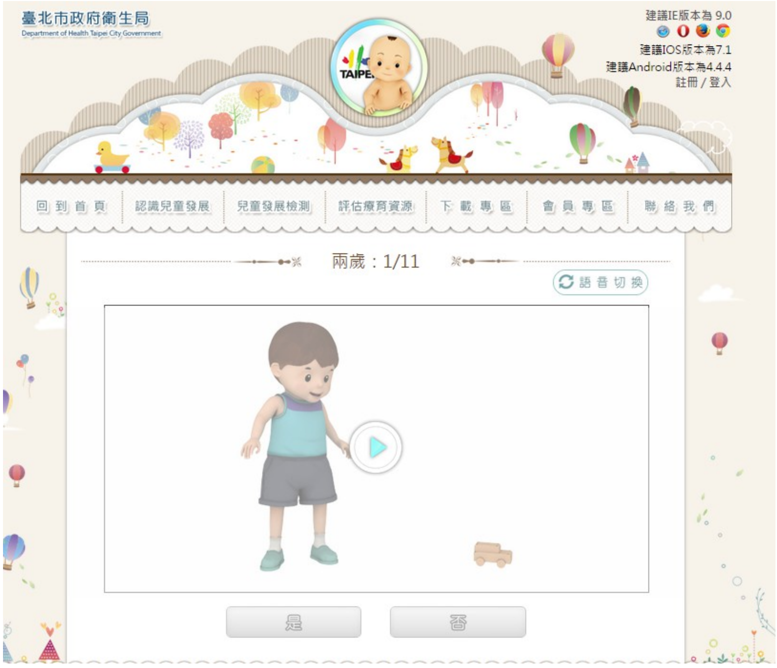

STEP6 Screening Completed, Upload the Results

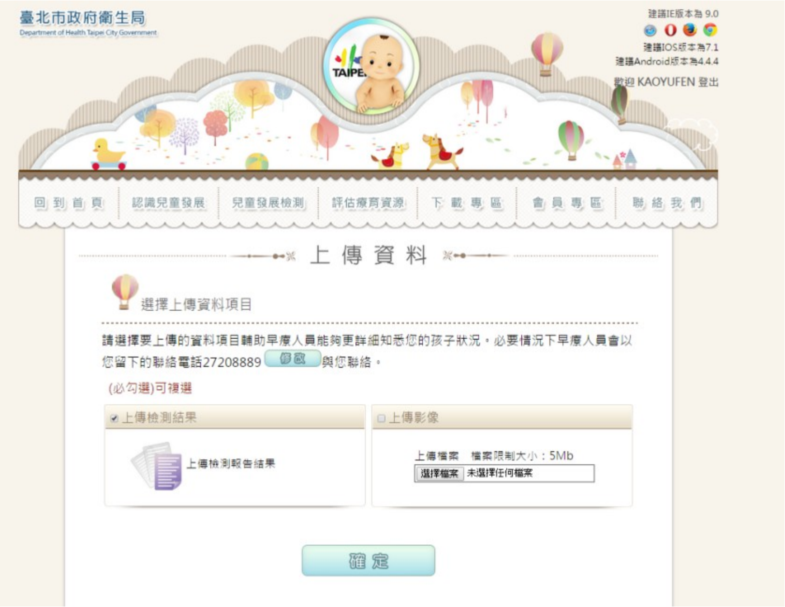

Upload File(s): Choose the file you want to upload

Choose the file(s) you want to upload. The information will help early intervention provider better understand your child. If needed, the provider will call you to the number you provide.

Upload Results, Upload an Image, Upload a File, File Size Limit: 5Mb

Developmental Screening for 0-6 Year Olds

◎What is Child Development?

A process of physical "growth" in height, weight and organs as well as "development", such as change, progress, maturity, of organ function, mental capacity and so on.

◎Understanding Developmental Delays in Children

In the process of growth and reaching maturity, various reasons (such as physiological, psychological, social or environmental factors) could lead to different degrees of delays or abnormalities in cognitive development, motor development(including muscle tone and balance), physiological development, language and communication development, psychological and social development, emotional development or self care skills.

◎Taipei City Child Developmental E-Screening Interactive Website

"Early Detection, Early Diagnosis and Early Intervention" is the best way to safeguard children's health. With lively and interesting audio, image and animation/interactive media design, Taipei City Child Developmental E-Screening Interactive Website (http://e-screening.health.gov.tw/) makes screening for children easy and simple. With the site, you can keep abreast of your child's new development. Once results have been uploaded, professional support will be provided in the case of possible atypical development. Early detection and treatment is extremely important to the golden period of child development.

How to Use Child Growth Charts

兒童生長曲線百分位圖

兒童生長曲線百分位圖包括身長／身高、體重與頭圍3種生長指標，分為男孩版和女孩版。生長曲線圖上畫有97、85、50、15、3等五條百分位曲線；百分位圖是在100位同月（年）齡的寶寶中，依生長指標數值由高而低、重而輕，從第100位排序至第1位。

兒童生長曲線圖的身長／身高圖，在2歲時的曲線有落差，主要是因為測量身長／身高的方法不同；2歲前是測量寶寶躺下時的身長，2歲後則是測量站立時的身高。

以1.5個月大體重5公斤的男寶寶為例：

1. 【年齡】1.5個月大向上延伸。
2. 【體重】5公斤重橫向延伸。
3. 在【年齡】與【體重】交會處，即A點。
4. 參照右方的百分位曲線數值，發現體重是【第50百分位】，代表在100名同年齡的男寶寶裡，其體重大約排在第50位。

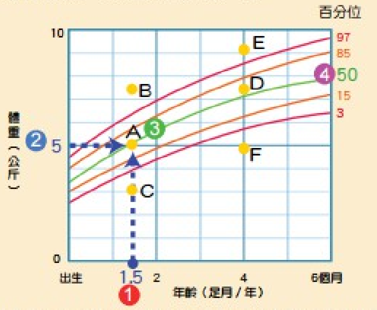

（請試著查查看3個月大男孩體重6.5公斤的百分位喔！答案請見下方）

寶寶的生長指標落在第3-97百分位之間都屬正常範圍，若生長指標超過第97百分位（如上圖B點）或低於第3百分位（如上圖C點）就可能有過高或低的情形！此外，兒童的成長是連續性的，除了觀察寶寶單一年齡的曲線落點外，其生長連線也應該要依循生長曲線的走勢（如上圖A點→D點）；如果高於或低於二個曲線區間時（如上圖A點→E點或A點→F點），需要請醫師評估檢查喔！

0-5歲生長曲線圖，係採世界衛生組織公布適用全球0-5歲兒童生長曲線標準圖；其以跨國合作方式，調查餵食母乳並在良好健康環境成長的兒童生長情形，故可避免將餵食母乳的嬰兒誤判為體重不夠的情形。

5-7歲生長曲線圖，係採用陳偉德教授2010年研究結果，以WHO 0-5歲生長曲線，銜接臺灣7-18歲依體適能訂定之生長標準，並參考WHO BMI rebound趨勢，據以繪製5-7歲生長曲線。

Translations:

Growth charts measure up your child in length/height, weight and head circumference against other children of the same gender. On the chart, there are 5 percentile curves with numbers of 97, 85, 50, 15 and 3. A percentile ranks the position of a baby in 100 babies of the same age. The 100th percentile curve stands for the highest, heaviest and largest, while the 1st percentile curve stands for the shortest, lightest and smallest.

The change in the length/height percentile curves at the age of 2 is the result of different measuring methods. Before the age of 2, we measure the lengths of babies in a lying position, and after the age of 2, we measure their heights in a standing position.

Take a 1.5-month-old, 5-kg baby boy for example:

(Age) Find 1.5 months and draw a vertical line.

(Weight) Find 5 kg and draw a horizontal line.

Find the spot these two lines cross each other, in this case, point A.

Follow the curve to the right and find his percentile. In this case, it is the 50th, which means his weight ranks approximately the 50th in 100 baby boys of the same age.

Weight (kg), Birth, Age (Month/Year), Month, Percentile

(Try to find the percentile of a 3-month-old, 6.5-kg baby boy and check with the answer below.)

Anything between the 3rd and 95th percentile is considered normal. Being above the 97th percentile (point B) or below the 3rd percentile (point C) may indicate an abnormality. In addition, child growth is a continual process. Besides the percentile at a certain age, you also need to follow your child's growth over time to find a pattern of his or her growth (ex. point A to point D). If your child moves up or falls down more than 2 curves (ex. point A to point E or point A to point F), please consult a doctor for evaluation and checkups.

Growth charts for 0-5 year olds are based on World Health Organization (WHO) growth standards for 0-5 year olds around the world. The standards describe normal breastfed child growth from birth to 5 years under optimal environmental conditions worldwide, and subsequently rule out the possibility of interpreting breastfed babies as underweight.

Growth charts for 5-7 year olds are based on Professor Walter Chen’s research in 2010. WHO growth standards for 0-5 year olds, Taiwan growth standers for 7-18 year olds based on physical fitness and WHO BMI rebound are all taken into consideration in designing the growth charts.

Growth Chart (Boy)

0-7歲生長百分位

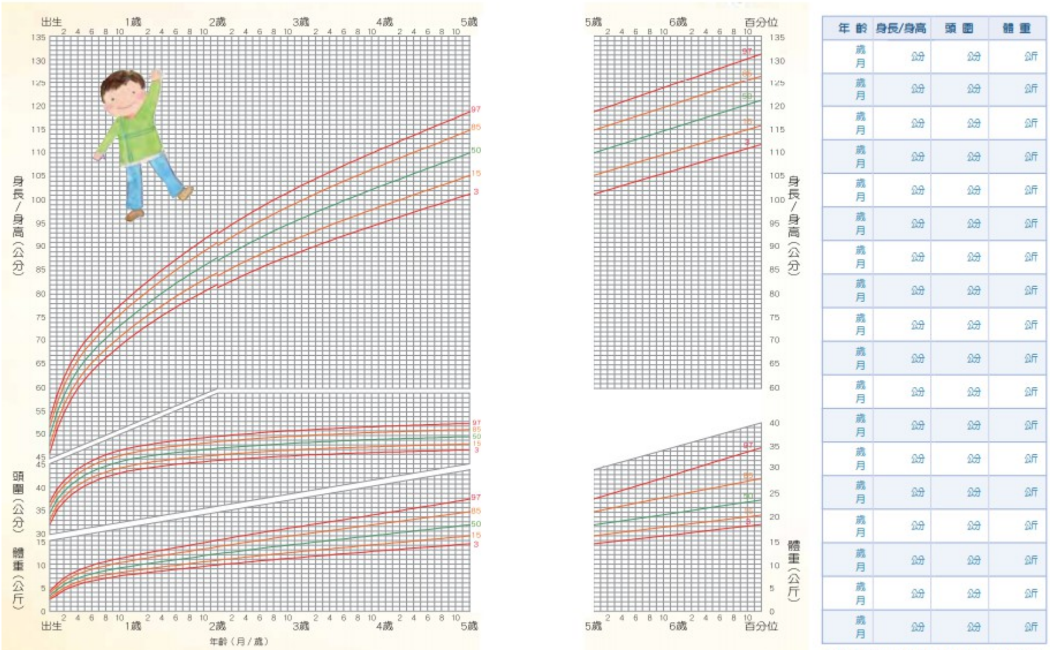

早產兒未滿3歲的年齡應自預產期起算(即矯正年齡)

Translations: Percentile for 0-7 Year Olds, Birth, 1 Year, 2 Years, 3 Years, 4 Years, 5 Years, 6 Years, Percentile

Length/Height (cm), Head Circumference (cm), Weight (kg), Age (Month/Year)

For premature infants, age should be calculated from the due date (corrected age)

Growth Chart (Girl)

0-7歲生長百分位

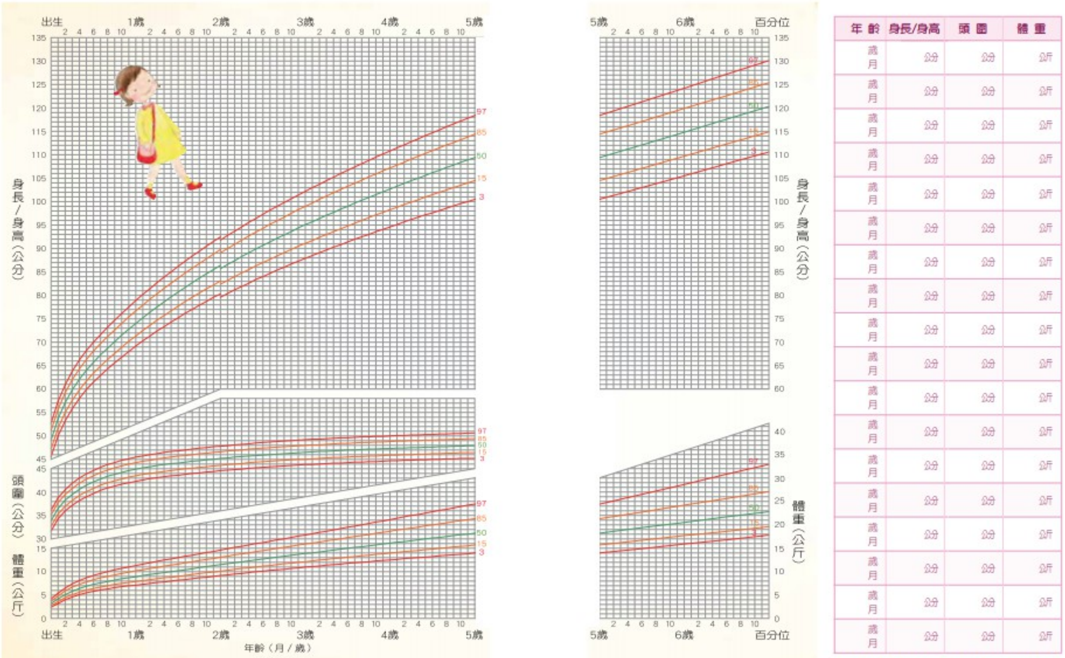

早產兒未滿3歲的年齡應自預產期起算(即矯正年齡)
